# Case Report: *STAT3* hyper-IgE syndrome in children: two cases report with uncommon complications of tuberculosis and lymphoma

**DOI:** 10.3389/fped.2026.1872306

**Published:** 2026-08-19

**Authors:** Xiaobei Cao, Bo Wang, Yongsheng Xu

**Affiliations:** Department of Pediatric Respiratory Medicine, Tianjin Children's Hospital (Children's Hospital, Tianjin University), Tianjin Key Laboratory of Birth Defects for Prevention and Treatment, Tianjin, China

**Keywords:** hyper-IgE syndrome, intestinal tuberculosis, lymphoma, miliary pulmonary tuberculosis, STAT3

## Abstract

**Background:**

Hyper-IgE syndrome (HIES) is a rare primary immunodeficiency disorder distinguished by a triad of eczema, recurrent skin and pulmonary infections, and elevated serum IgE levels. The loss-of-function mutations in signal transducer and activator of transcription 3 (*STAT3*) result in STAT3-HIES, which is considered the prototype form of HIES. Patients with STAT3-HIES are susceptible to *Staphylococcus aureus* and *Candida albicans*, while *Mycobacterium tuberculosis* infection is rare, usually accompanied by multiple non-immunologic features including skeletal and connective tissue abnormalities, and the increasing risk of lymphoma. Owing to the diverse clinical manifestations of this disease and the lack of specific symptoms in its early stage, delayed diagnosis often occurs.

**Case presentation:**

This report describes two cases of children with STAT3-HIES. One case was a 3-year-old child who presented with intestinal intussusception as the initial symptom and was considered to have probable intestinal tuberculosis and accompanied by miliary pulmonary tuberculosis, and tuberculosis was cured after one year of anti-infection treatment, HIES combined with intestinal tuberculosis presenting with intussusception as the clinical manifestation has never been reported. Second case was a 16-year-old child who was diagnosed with anaplastic lymphoma kinase-negative anaplastic large cell lymphoma because of swollen lymph nodes in the neck at the age of 10, he underwent allogeneic hematopoietic stem cell transplantation when he was 16 years old, unfortunately he died of lung infection after three months.

**Conclusion:**

The cases described in this article enhance our understanding of the manifestations, treatment and prognosis of this syndrome, highlighting the hazards of active tuberculosis and lymphoma in patients with HIES, as well as the importance of timely diagnosis, individualized treatment and follow-up.

## Introduction

Hyper-IgE syndrome (HIES) is a rare primary immunodeficiency disorder characterized by eczema, recurrent skin and lung infections along with elevated serum IgE levels. The disease usually begins during early childhood. Most cases are sporadic, familial cases with autosomal dominant (AD), autosomal recessive (AR), and rare X-linked inheritance patterns have also been reported. Classic HIES is primarily caused by loss-of-function mutations in the signal transducer and activator of transcription 3 (*STAT3*), while defects in *ERBIN*, *IL6ST*, and *CARD11* can also lead to an AD-HIES phenotype; the most common cause of AR-HIES is *DOCK8* deficiency, with other causative genes including *PGM3* and *TYK2,* while *DOCK8* deficiency has been reclassified as a combined immunodeficiency according to the latest phenotypical classification of International Union of Immunological Societies (IUIS) (1–5). Although various HIES subtypes exhibit overlapping phenotypes, they differ substantially in infection spectrum, multisystem organ involvement, malignancy risk and therapeutic approaches. *STAT3*, a member of the JAK-STAT pathway, is a transcription factor in signal transduction pathways of multiple cytokines, especially involved in IL-6, IL-10 and IL-23 (6, 7). These pathways play critical roles in regulating immune and non-immune cell development and function. Aberrant signaling through these pathways contributes to the multisystem manifestations of STAT3-HIES, including immunologic features such as recurrent skin and lung infections, as well as non-immunologic manifestations including characteristic facial features, skeletal and connective tissue abnormalities, even lymphoma. Defective STAT3 signaling results in impaired Th17 cell differentiation and insufficient IL-17 secretion, which explains the increased susceptibility to *Staphylococcus aureus* and *Candida albicans* in the skin and respiratory tract (8). Patients with *DOCK8* deficiency exhibit severe impairment of NK cell and T cell function; in addition to bacterial and fungal infections, they are more vulnerable to refractory cutaneous viral infections (e.g., herpes simplex virus, human papilloma virus), and have a high prevalence of malignancies, food allergies and intestinal inflammation, whereas central nervous system complications are relatively rare. *PGM3* deficiency is classified as a congenital disorder of glycosylation; in addition to manifestations such as recurrent infections and skeletal abnormalities, patients frequently present with neurological features including developmental delay, intellectual disability, and hypotonia. Dominant-negative mutations in *CARD11* disrupt NF-κB signaling and cause a distinct atopic disorder characterized by severe eczema, asthma, food allergies, and frequent respiratory and cutaneous viral infections. *TYK2* deficiency directly disrupts the IL-12/IFN-γ anti-mycobacterial pathway, rendering patients susceptible to tuberculosis and Bacillus Calmette-Guérin (BCG)-induced disease, this subtype of HIES exhibits the highest prevalence of mycobacterial infections (9). The fundamental treatment strategies include active infection prevention and standardized skin care, together with appropriate administration of targeted biologic agents. Immunoglobulin replacement therapy can reduce infection risk and is particularly beneficial for patients with antibody deficiency (10, 11). However, recurrent pulmonary infections may still occur and progress to parenchymal lung lesions. Frequent pneumatocele formation is pathognomonic for STAT3-HIES, and together with bronchiectasis, these lesions drive disease-related morbidity and mortality (12). Furthermore, the incidence of malignancies rises with advancing age, which severely impairs patients’ quality of life and survival, particularly among individuals with STAT3-HIES and *DOCK8* deficiency (13). Hematopoietic stem cell transplantation (HSCT) serves as a vital therapeutic modality, it carries curative potential but is accompanied by limitations. In this report, we describe the manifestations and treatment outcomes of a STAT3-HIES child with probable intestinal tuberculosis presenting as intussusception and complicating miliary pulmonary tuberculosis, and another STAT3-HIES child with anaplastic lymphoma kinase (ALK)-negative anaplastic large cell lymphoma(ALCL).

## Case presentation

### Case 1

**Figure 1:**
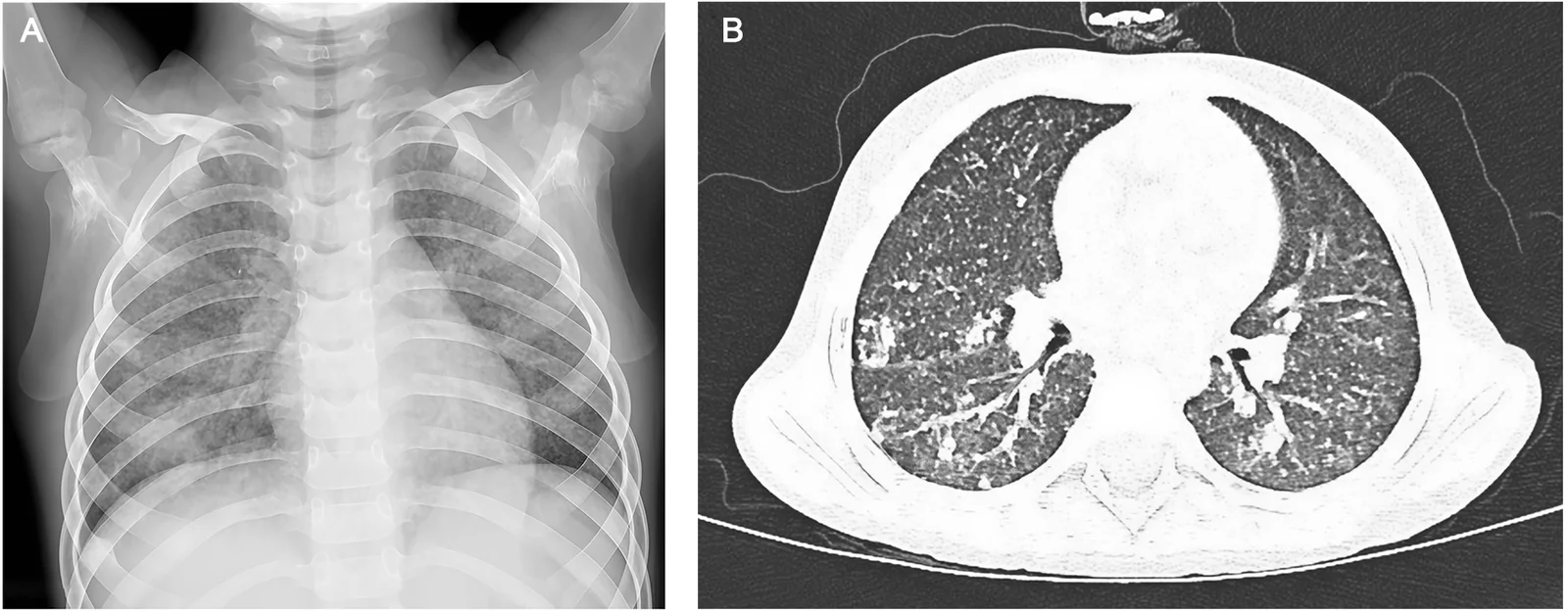
Chest radiograph and computed tomography (CT) of the patient. **(A)** Chest radiograph shows diffuse distribution of point-like and bilateral patchy high-density shadows. **(B)** Chest CT demonstrates diffuse multiple miliary and bilateral nodular and scattered patchy shadows.

**Figure 2:**
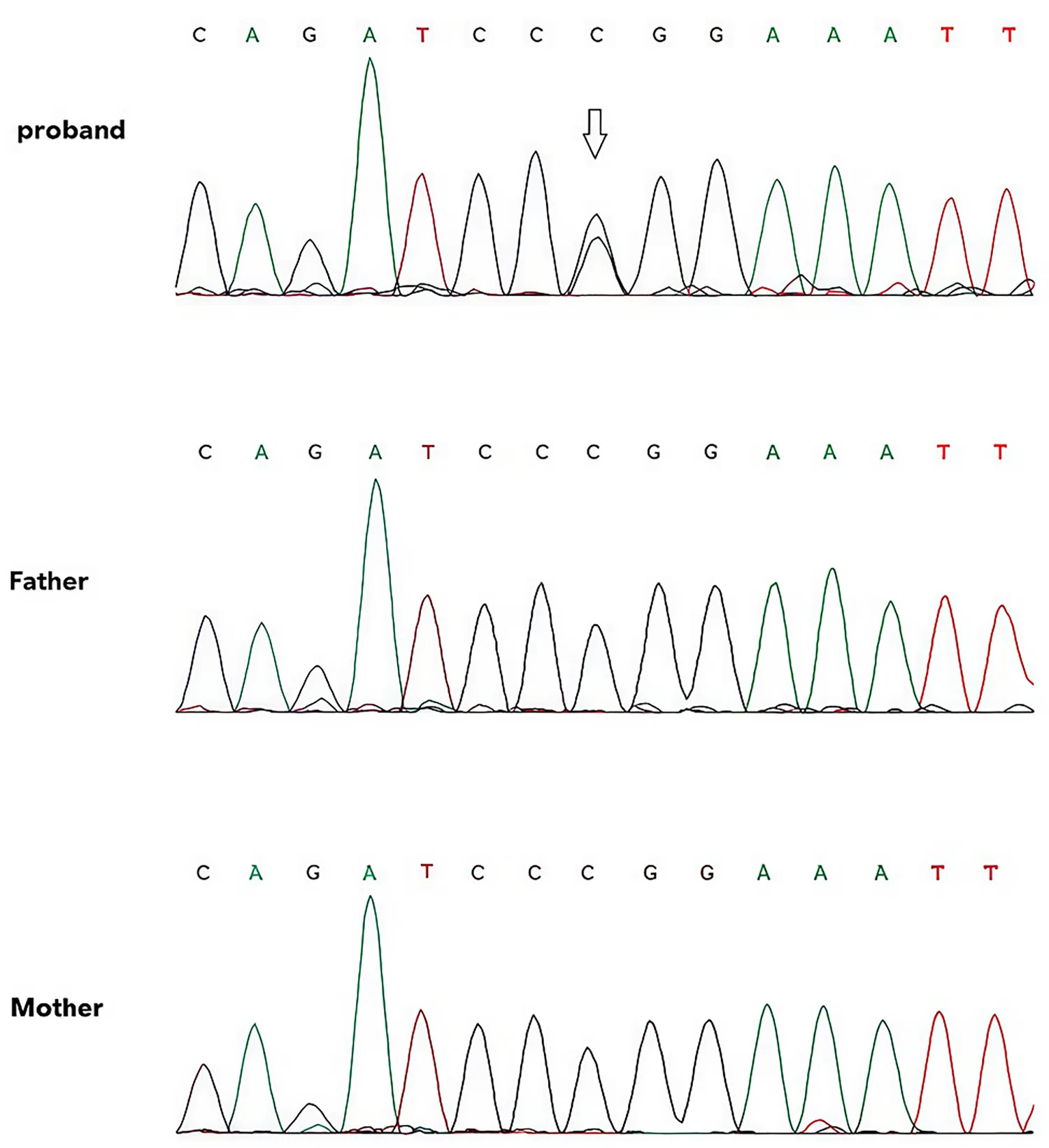
Sanger sequencing chromatograms of the *STAT3* c.1144C > G (p.Arg382Gly) variant in P1. The proband exhibits a heterozygous overlapping C/G peak at the indicated position, while both parents show homozygous wild-type C peaks, confirming a *de novo* heterozygous mutation.

The Chinese patient (P1) was a 3-year-old boy when he was admitted to the hospital because of abdominal pain and vomiting for one day. He was the first child born to healthy non-consanguineous parents with no family history of genetic diseases. His past medical history included recurrent eczema, chronic oral and cutaneous candidiasis, and skin abscesses due to *Staphylococcus aureus*, all of which had been present since birth. He also developed pneumonia, sepsis, and otitis media at the age of 2 months. Physical examination showed that the child was at appropriate age of growth and development, BCG scar was absent (the child received standard BCG vaccination at birth but failed to form a visible scar). He had rough skin all over the body, scattered papules on the trunk, high palate, and characteristic facial features with prominent forehead, broad nasal bridge and thick lips. The abdominal ultrasound indicated intussusception and failed to reduce after two attempts of air enema, then exploratory laparotomy and manual reduction of intussusception were performed. Fever and cough developed two days postoperatively, and chest radiography demonstrated bilateral diffuse pulmonary opacities (Figure 1A). Subsequently, chest CT scan revealed diffuse and multiple granular-like small nodules in both lungs, along with scattered inflammatory infiltrates (Figure 1B). The tuberculin skin test and interferon-gamma release assays were negative, which may be explained by young age, severe disseminated mycobacterial disease, immune anergy, or underlying IL-12/IFN-γ pathway defects. The IgE was 37,533 IU/mL (reference range: < 100 IU/mL), absolute eosinophil count was 0.74 × 10^9^/L. The metagenomic next-generation sequencing (mNGS) of bronchoalveolar lavage fluid (BALF) detected *Mycobacterium tuberculosis* (45 reads) after fiberoptic bronchoscopy, the GeneXpert MTB/RIF test of BALF showed positive MTB-DNA and negative rifampicin resistance gene, while mycobacterial culture was negative, a diagnosis of miliary pulmonary tuberculosis was established accordingly. Intraoperatively, annular ulcers and nodules were visualized at the ileocecum, with thickening of the proximal 80 cm of intestinal wall, but no intestinal histopathological biopsy was obtained at that time. Abdominal ultrasound showed multiple thickening of intestinal wall, the GeneXpert MTB/RIF test of stool sample showed positive MTB-DNA and negative rifampicin resistance gene. Based on these findings, intestinal tuberculosis was clinically suspected. Trio whole-exome sequencing (trio-WES) combined with Sanger validation identified a heterozygous *de novo STAT3* (c.1144C > G, p.Arg382Gly) variant absent in both parents (Figure 2), located in the *STAT3* DNA-binding domain. Sequencing parameters: mean WES depth >120 ×, 154 × locus coverage, 49.4% VAF; reads were aligned to UCSC hg19 via BWA, genomic coordinate chr17:40481661, reference transcript NM_139276.3. This variant is classified as pathogenic under the 2015 ACMG/AMP guidelines. HGMD records the pathogenic variant c.1144C > T (p.Arg382Trp) at the same codon, and c.1144C > G (p.Arg382Gly) has been documented in STAT3-HIES patients. Trio-WES fully excluded deleterious variants in MSMD-related genes, including *IFNGR1*, *IFNGR2*, *IL12RB1*, *CYBB*, and *IKBKG/NEMO*, the patient was ultimately diagnosed with STAT3-HIES (Table 1). He was treated with Rifampin, Isoniazid, Pyrazinamide, and Ethambutol for one year, and tuberculosis was cured through outpatient follow-up. One year later, at 4 years of age, the child was hospitalized again because of fever and cough. Chest CT revealed scattered inflammatory consolidations in both lungs, atelectasis of the right upper lobe, and right pleural effusion. Bronchoscopy demonstrated inflammatory changes in the bronchial mucosa of the right lung, along with yellowish-white secretions at the opening of the right upper lobe bronchus. The mNGS of BALF identified Mycoplasma pneumoniae infection (134,868 reads) without evidence of *Mycobacterium tuberculosis*, and GeneXpert MTB/RIF testing of BALF was negative. The patient recovered and was discharged after one week of hospitalization. The patient has been followed up until now, when he is 6 years old, and no serious infections occurred again. The child received oral antibacterial and antifungal prophylaxis throughout follow-up, while regular intravenous immunoglobulin replacement was not administered due to parental refusal and stable clinical condition.

**Table 1:** Diagnostic evaluation of two patients with STAT3-HIES using the NIH scoring system and woellner criteria.

Diagnostic assessment tool	Parameters	P1 (3 years old)	P2 (16 years old)
NIH-HIES score	Maximum serum IgE (IU/mL)	10	10
	Eczema	4	4
	Recurrent skin abscesses	4	4
	Recurrent pneumonia	6	8
	Permanent lung lesions	0	8
	Candidiasis	4	4
	Retained primary teeth	0	2
	Scoliosis	0	2
	Fractures	0	4
	Characteristic facial features	4	4
	High-arched palate	2	2
	Joint hyperextensibility	0	4
	Newborn rash	4	4
	Eosinophilia	3	6
	Other severe infections	4	4
	Lymphoma	0	4
	Total NIH score (points)	45	74
Woellner criteria	Possible HIES (IgE>1,000 +core features)	Met	Met
	Probable HIES (family history/reduced Th17)	Not fully met	Not fully met
	Definitive HIES (pathogenic *STAT3* variant)	Met	Met

### Case 2

The Chinese patient (P2) was a 16-year-old boy who was admitted to our hospital due to dyspnea for one day. He was the second child born to healthy non-consanguineous parents with no family history of genetic diseases. He suffered from persistent eczema since birth, accompanied by recurrent skin and pulmonary infections caused by *Staphylococcus aureus* and *Candida albicans*, and also experienced pathological fractures after minor trauma. Physical examination revealed generalized scattered rashes and rough skin, alongside classic disease phenotypes: distinctive facial appearance, a high-arched palate, retained primary teeth, scoliosis, and hyperextensible joints of the extremities. At the age of 9 years and 10 months, he was hospitalized due to acute gastroenteritis and sepsis. During this period, bone marrow aspiration was performed without any abnormalities, However, his IgE > 2,500 IU/mL (reference range: < 100 IU/mL) and absolute eosinophil count was 0.9 × 10^9^/L. At the age of 10, he developed a a right neck mass, ultrasound showed bilateral cervical lymphadenectasis with the maximum size of approximately 41 mm × 19 mm. Core needle biopsy of the right cervical mass revealed the following immunophenotype: CD3 (+), CD20 (−), CD30 (+), partial CD4 (+), partial CD8 (+), ALK (−), EMA (−), Granzyme B (−), Ki-67 (80%+), and EBER (−). Due to limited tissue obtained from the biopsy and atypical immunophenotype, the specimen was sent to Peking University Pathology Center, a final diagnosis of ALK⁻ ALCL was established. Unfortunately, pathological micrographs, TCR clonality assay and fusion gene testing were not accessible. Meanwhile, genetic testing showed heterozygous *de novo* mutation in *STAT3*, but no abnormalities were detected in his parents, the patient was diagnosed as STAT3-HIES (Table 1). He was given chemotherapy with CTX + Dex and AA-BB-CC-AA-BB-CC regimens for six cycles, and the last chemotherapy was at the age of 10 years and 8 months, the efficacy evaluation of PET-CT was close to complete remission. During follow-up, the child was hospitalized due to recurrent pneumonia and gastrointestinal infections. At the age of 16, the PET-CT showed changes after lymphoma treatment, multiple lymph nodes were found in the submental space, carotid space and posterior cervical space, and some were slightly enlarged with slightly increased metabolism, also multiple small lymph nodes in both axillae and groin with low metabolism, and diffuse increased metabolism in the bone marrow cavity, moreover, multiple ground-glass opacities and patchy shadows were observed in the upper lobe of right lung and the lower lobes of both lungs, and multiple irregular air-filled cavities with thick walls in the posterior segment of left lung, suggesting secondary infection due to bronchiectasis. At 16 years and 6 months of age, he received allogeneic peripheral blood stem cell transplantation with an 8/10 HLA-matched donor at an outside hospital, granulocyte implantation was alive after 14 days, but platelet was not implanted. One-month post-HSCT, severe pulmonary infection occurred. The chest CT scan showed consolidation shadows, ground-glass changes, cavity shadows, bullae of lung, pleural effusion, decreased bone density of the sternum components, and shortening of the high columns of some centrum in thoracic vertebrae. Two months post-HSCT, the child progressively developed dyspnea and hypoxemia and required mechanical ventilation. After being transferred to our hospital, the re-examination of imaging was consistent with pneumocystis pneumonia. Bronchoscopy could not be performed due to unstable vital signs, and confirmatory tests including Pneumocystis PCR, serum beta-D-glucan assay and induced sputum examination were unavailable, the diagnosis was therefore established solely based on clinical and imaging findings. The patient subsequently developed multiple organ failure including renal dysfunction, and received supportive treatment such as CRRT. Three months post-HSCT, the patient ultimately died of respiratory and circulatory failure, acute respiratory distress syndrome and severe pneumonia.

## Discussion

The hallmark immunological defect of STAT3-HIES lies in dysfunction of the canonical phosphorylated JAK-STAT signaling pathway, which disrupts Th17/IL-17 responses, thereby resulting in susceptibility to extracellular bacteria and fungi (1, 8). The natural defense ability of human to the mycobacteria group depends on the IL-12/IFN-γ pathway, and functional defects in this pathway represent the classic pathogenic mechanism of Mendelian susceptibility to mycobacterial disease (MSMD), individuals with this type of immunodeficiency are highly susceptible to weakly virulent mycobacteria (e.g., BCG and environmental mycobacteria) and *Mycobacterium tuberculosis* (14). Accordingly, this isolated impairment of the Th17/IL-17 axis cannot fully account for the miliary pulmonary tuberculosis observed in P1. Sporadic cases of active tuberculosis in HIES patients have been reported over the past 20 years (Table 2) (15–19). A national study in India retrospectively analyzed 103 cases with clinical and/or molecular diagnosis of STAT3-HIES, the overall incidence of *Mycobacterium tuberculosis* infection was 5.8% (18). Although the incidence of tuberculosis infection in STAT3-HIES patients is relatively low, and the mechanisms underlying susceptibility to tuberculosis remain incompletely elucidated, STAT3-HIES and MSMD share critical overlapping JAK-STAT signaling pathway that is indispensable for immune regulation and host defense against intracellular pathogens (20). *STAT1* and *STAT3* both belong to the JAK downstream transcription factor family. MSMD is characterized by impaired STAT1-dependent IL-12/IFN-γ responses, whereas loss-of-function mutations in *STAT3* interfere with signal transduction in this pathway, thereby attenuating the mycobactericidal capacity of the host. In recent years, Karim et al. described impairment of the unphosphorylated STAT3-unphosphorylated NF-κB (uSTAT3-uNF-κB) pathway in loss-of-function STAT3-HIES (21). This defect downregulates critical effector molecules including *RANTES* and *STAT3,* which impairs immune defense function and raises the risk of infections caused by bacteria, fungi, viruses, and even mycobacteria. A single-center clinical study of adults in China proposed that recurrent pulmonary infections cause structural lung damage, thereby increasing the pulmonary susceptibility and providing an opportunity for *Mycobacterium tuberculosis* infection (19). Furthermore, Zhang et al. suggested that *STAT3* mutations lead to defects in the host defense function and bacterial clearance ability of airway epithelial cells, which may also cause pulmonary infection with *Mycobacterium tuberculosis* (22). Therefore, the possible mechanisms underlying tuberculosis susceptibility in STAT3-HIES patients include: dysfunction of the IL-12/IFN-γ pathway, systemic immune dysregulation mediated by the uSTAT3–uNF-κB pathway, impaired airway epithelial defense and lung structural damage resulting from recurrent pulmonary infections. If STAT3-HIES patients are diagnosed with severe tuberculosis, concurrent screening for IL-12/IFN-γ pathway defects and MSMD should be performed.

**Table 2:** The information of complicated tuberculosis in HIES patients over the past 20 years.

Number	Article, Publication year	Country	Age (years)	Types of tuberculosis
1	Metin et al., 2004 (15)	Turkey	7	tuberculous brain abscess
2	Mahla, 2012 (16)	India	20	pulmonary tuberculosis
3	Singhal et al., 2013 (17)	India	6	Tuberculous osteomyelitis
4	Saikia et al., 2021 (18)	India	median 5	Pott's spine, pulmonary tuberculosis
5	Xie et al., 2025 (19)	China	median 28.1	pulmonary tuberculosis
6	This article, 2026	China	3	intestinal tuberculosis,miliary pulmonary tuberculosis

For Nos. 1 and 3, STAT3-HIES was clinically suspected; No. 2, *STAT3* was positive by polymerase chain reaction based on high resolution DNA melting assay; No. 4 refers to a cohort of six STAT3-HIES patients including two genetically confirmed STAT3-HIES and four clinically diagnosed cases (median age 5 years); No. 5 includes five genetically confirmed STAT3-HIES patients with a median age of 28.1 years; No. 6 corresponds to the genetically confirmed STAT3-HIES patient reported in this study.

Previously reported cases of active tuberculosis in STAT3-HIES patients show multisystem involvement, including the skin, lungs, bones and central nervous system; however, intestinal tuberculosis has not been documented. Intestinal tuberculosis often occurs in the ileocecal region, the main route of infection is ingestion of sputum containing *Mycobacterium tuberculosis* or infected milk by patients, secondary routes of infection include hematogenous spread to the intestine from miliary pulmonary tuberculosis, as well as direct spread from tuberculous lesions within the abdominal and pelvic cavities (23). *Mycobacterium tuberculosis* preferentially colonizes lymphoid tissue within the ileocecal region, forming characteristic annular ulcers and tuberculous granulomas, accompanied by intestinal wall thickening, fibrous proliferation and cicatricial contraction; these pathological changes cause intestinal luminal stenosis, which can further progress to intestinal obstruction or even intussusception (23, 24). Definite diagnosis of intestinal tuberculosis relies on combined histopathological and microbiological tests of intestinal biopsy specimens. The identification of caseating granulomas on histology strongly supports the diagnosis, while detection of acid-fast bacilli in tissue, positive mycobacterial culture, or positive GeneXpert MTB/RIF assay enables definitive etiological confirmation (25). P1 presented with intussusception as the initial manifestation, intraoperative inspection revealed annular ulcers with nodules localized to the ileocecum, accompanied by bowel wall thickening, the GeneXpert MTB/RIF test of stool specimen showed positive MTB-DNA. Since there were no obvious respiratory or chronic gastrointestinal symptoms before onset of the disease, intestinal tuberculosis was not considered in early stage, and no histopathological examination was conducted, thus pathological diagnostic evidence was lacking. Moreover, a positive GeneXpert result from stool specimen could not exclude the possibility of false positivity due to swallowed sputum containing *Mycobacterium tuberculosis*. With confirmation of miliary pulmonary tuberculosis, intestinal tuberculosis is clinically suspected in this patient. Notably, intussusception in this setting is not directly induced by *STAT3* pathway abnormalities, but rather occurs as a secondary complication of intestinal tuberculosis. We summarize global cases of HIES combined with tuberculosis over the past two decades, confirming that tuberculosis constitutes a severe infectious complication of STAT3-HIES. In countries with a high burden of tuberculosis, *Mycobacterium tuberculosis* should be incorporated into the primary screening pathogen panel for STAT3-HIES patients in addition to the classic pathogens *Staphylococcus aureus* and *Candida albicans*.

Apart from recurrent infectious complications, lymphoma constitutes a critical long-term adverse prognostic factor in patients with STAT3-HIES. Previous studies have demonstrated that the incidence of malignancies in HIES patients was 6.5%, with significantly higher rates in patients carrying *STAT3* or *DOCK8* mutations, diffuse large B-cell lymphoma is the predominant subtype in STAT3-HIES, whereas T-cell anaplastic large cell lymphoma is extremely rare (13, 26, 27). At present, there are two reports on HIES combined with ALCL. One case of STAT3-HIES patient was diagnosed with ALK⁻ ALCL at the age of 21 (28), another patient was diagnosed as HIES at the age of 22, she was pathologically diagnosed with ALCL due to cervical lymphadenectasis at the age of 56 (29). Lymphomagenesis in STAT3-HIES patients is driven by multiple synergistic factors: *STAT3* mutations cause defective Th17 differentiation and impaired function of NK and CD8 ^+^ T cells, attenuating the host's capacity to eliminate mutated cells and enabling tumor cells to evade immune attack; recurrent chronic cutaneous and pulmonary infections induce a chronic inflammatory microenvironment that persistently stimulates aberrant lymphocyte proliferation; most importantly, *STAT3* acts as an oncogenic transcription factor, its loss-of-function directly disrupts signaling pathways governing lymphocyte proliferation and apoptosis, thereby driving malignant transformation of lymphocytes; recent studies have confirmed that disorders in the uSTAT3-uNF-κB pathway can further amplify inflammatory and carcinogenic signals (21, 30–33). Regarding the median age at onset of lymphoma in patients with STAT3-HIES, a Chinese study reported a median age of 34 years, while a French national survey recorded 21 years (27, 28). P2 was diagnosed as ALK⁻ ALCL at the age of 10, which is younger than the median age of onset aboved, suggesting that recurrent infections at early childhood may have accelerated the process of lymphoma.

The indications and outcomes of HSCT differ substantially between STAT3-HIES and *DOCK8* deficiency. HSCT is curative in *DOCK8* deficiency (34), while the therapeutic effect on STAT3-HIES is uncertain (35). A worldwide multicenter retrospective study analyzed HSCT in 41 STAT3-HIES patients over a 28-year period (36). The study demonstrated that the skin and respiratory diseases of patients were improved after HSCT, but the impact on non-immunological manifestations such as skeletal lesion seemed to be limited, also reported 4 cases of death after transplantation including 1 case of implantation failure; the study also indicated that the indications for HSCT comprised recurrent severe infections, parenchymal lung disease or lymphoma, and the median age at transplantation was 14 years, with a 5-year overall survival rate of 93%. P2 presented with recurrent severe infections, progressive lung disease and ALK⁻ ALCL, which met the core indications for HSCT in STAT3-HIES. Unfortunately, he ultimately died of pulmonary complications after transplantation at the age of 16. Therefore, although the overall survival of HSCT in STAT3-HIES is high, severe complications or death caused by implantation failure may still occur in individual cases, suggesting that individualized treatment needs to be further optimized in order to improve the prognosis of children.

Despite the novel clinical insights provided by the two rare cases in the present study, several inherent limitations should be acknowledged. First, the sample size is restricted to only two pediatric STAT3-HIES patients, so the observational findings lack sufficient generalizability, and larger multicenter prospective cohorts are warranted to externally validate our clinical observations. Second, we failed to obtain intestinal biopsy samples from P1, which prevented histopathological confirmation of intestinal tuberculosis; moreover, functional immune examinations, such as Th17 cell enumeration, IL-17A/IL-22 secretion detection, and phospho-flow cytometry detecting cytokine-induced *STAT3* phosphorylation, were not carried out to further verify underlying immune defects. Third, complete pathological data of the lymphoma lesion in P2 and full in-depth genetic characterization of the identified *STAT3* pathogenic variant were not accessible in this research. Future multi-center stratified studies are needed to identify optimal candidates and the best timing for curative interventions including HSCT, so as to build standardized precision management strategies for pediatric STAT3-HIES patients with high-risk complications.

## Conclusion

This study reports the first case of STAT3-HIES presenting with intussusception as the initial manifestation, complicated by miliary pulmonary tuberculosis and clinically suspected intestinal tuberculosis, which highlights the severe infectious risk of tuberculosis in patients with this immunodeficiency disorder. The second patient developed ALK⁻ ALCL at the age of 10, far younger than the reported median age of lymphoma onset among STAT3-HIES populations, which indicates that early regular tumor surveillance is necessary for affected children. Targeted individualized intervention and standardized long-term follow-up should be established for high-risk children complicated with severe infection or malignant tumors to reduce life-threatening complications and improve long-term survival outcomes.

## Data Availability

The data analyzed in this study is subject to the following licenses/restrictions: No raw clinical datasets are included in the manuscript or Supplementary Materials; only summarized clinical findings are presented. Requests to access these datasets should be directed to XC, xiabeicao@163.com.
